# Liquid biopsy. A challenge for clinical laboratories

**DOI:** 10.1515/almed-2020-0055

**Published:** 2020-07-27

**Authors:** Wladimiro Jiménez

**Affiliations:** Servicio de Bioquímica y Genética Molecular, Centro de Diagnóstico Biomédico, Hospital Clínico, Facultad de Medicina, Universidad de Barcelona, Barcelona, Spain

**Keywords:** DNA, liquid biopsy, next-generation sequencing, PCR

In the last years, clinical laboratories have faced numerous scientific, technological and digital challenges. This phenomenon has been parallelled by a continuous upgrading of the faculty ruling these facilities. This editorial intends to draw the attention of our readers to a new subject which is widely spreading within our professional community, the so-called liquid biopsy, a definition which may, at first, seem to be confusing.

The term liquid biopsy is widely used in oncology diagnostics and has been popularized by the *in vitro* diagnostics industry. This technique is applied to determine the characteristics of a tumour based on the analysis of circulating free DNA in blood. Although the origin of this DNA is not precisely known, it is known to originate in tumour and non-tumour cells that do not necrotize or undergo processes of apoptosis and release DNA to the bloodstream. In oncologic patients, about 50% of circulating DNA corresponds to small tumour DNA fragments [1]. Concentrations of up to 0.2 µg/mL have been documented in patients with advanced cancer, leading to the question of why liquid biopsy has raised and continues to raise such high expectations. Conventional biopsy is invasive, with tissue being limited or even inaccessible, often hindering the determination of tumour heterogeneity. However, the advantages of liquid biopsy are that it is minimally invasive, less costly, it is an indicator of tumour heterogeneity, and in some cases, liquid biopsy may be useful for monitoring patient response and/or resistance to treatment [2]. However, although it is evident, it should be taken into account that liquid biopsy is nothing more than another blood sample to be processed in clinical laboratories.

Liquid biopsy is mainly used in the diagnosis of oncological diseases and is performed using circulating free DNA from tumour cells. Specific alterations, translocations and methylation modifications can be detected in this DNA. However, it should be noted that liquid biopsy is not only used in the oncological setting. The use of liquid biopsy in non-invasive prenatal diagnosis may also be of great importance from a healthcare and social point of view, Circulating free DNA can be detected in foetal peripheral blood after the first trimester of pregnancy, making this a very important prenatal diagnostic technique [3], [4]. In addition, liquid biopsy has none of the disadvantages involved in obtaining amniotic fluid or chorionic villus sampling.

The analysis of circulating free DNA for diagnostic purposes in the oncological setting is complex as shown on examination of the characteristics of the cells involved in tumour transformation. The activity of oncogenes and tumour suppressor genes determines whether a specific cell transforms into a cancerous cell. An imbalance between these two factors can result in uncontrolled cell growth, inhibition of apoptosis, induction of angiogenesis and ultimately, cell invasion and metastasis. There are three general categories of oncogenes: tyrosine kinase receptors, growth factors and transcriptional activation factors. On the other hand, tumour suppressor genes may act as cell cycle regulators or inhibitors of proliferation.

Oncogenes are found in many cellular signalling pathways, which are controlled by tyrosine kinase receptors. The interaction of these membrane receptors with growth factors, cytokines or hormones activates the corresponding signalling pathway. Some examples of tyrosine kinase receptors include epithelial growth factor receptor, vascular growth factor receptor, and the fibroblast receptor. One of the most widely preserved signalling pathways in eukaryote cells is the Ras-Raf-Mek-Erk pathway. In the presence of a specific agonist such as a growth factor, the tyrosine kinase receptor sends a phosphorylation signal that activates this pathway, triggering a cascade of biological events including cell growth, survival and proliferation or differentiation. Many of the analytical targets analysed in liquid biopsy focus on the alterations in the genes involved in the regulation of these mechanisms

In addition to determining whether the mutations detected correspond to an oncogene or a tumour suppressor gene, there are also different situations based on the type of alteration a mutation carries. Thus, there may be variations in a single nucleotide, insertions or genomic fusions and variations in the number of copies. These are a few examples reflecting the extraordinary complexity of molecular diagnosis in oncological diseases. In addition, it should be taken into account that oncologic diseases are a dynamic process, and the status of a tumour may be assessed at different stages of its evolution such as when the first neoplastic alterations occur, in circumstances of tumour heterogeneity, or during treatment showing response with remission or in tumour recurrence. These different situations can also be seen as changes in circulating free DNA. It is not surprising that the overwhelming amount of data provided by liquid biopsy has raised some reluctancy among technicians to take on this technique in more conventional clinical laboratories. In any case, this issue of *Advances in Laboratory Medicine* includes an excellent review by Dr. María Arechederra et al. for readers interested in more in depth information on these concepts [5].

At this point, we would like to point out the main analytical approaches and instruments currently available. There are essentially two differentiated analytical strategies. One is used for evaluating well defined mutations and is known as targeted analysis. It is based on the use of real-time PCR in multiplex system which can be associated or not with subsequent sequencing and with the corresponding bioinformatic analysis. It should be highlighted that, at present, specific instruments and the corresponding kits are available for this technology. The second strategy is based on next generation sequencing. This technique enables the detection of known and unknown mutations following a non-targeted diagnostic strategy. In contrast with targeted analysis, this approach is not based on the use of specific kits for determined mutations. The workflow begins with the use of an automatic DNA extractor, followed by the implementation of manual and automated library preparation systems, depending on the amount of samples to be analysed and the ultra-sequencing system used [6]. Naturally, bioinformatics – which may include in-house arrays – are also applied [7]. Likewise, we should also mention the systems developed for specific applications such as first trimester screening in pregnant women, which consist of the analysis of circulating free foetal DNA in maternal blood [4]. It is important to note that alternative technologies are currently available and can be adapted to different diagnostic needs, with an elevated level of automatisation that is very likely to increase in the near future.

Another aspect of paramount importance is the economic impact that molecular diagnostics will have on clinical laboratories. Estimations indicate that the number of liquid biopsy tests performed in the USA will increase 10-fold within the next 10 years [8]. These estimations are justified by the fact that molecular diagnosis enables the use of selective therapies with demonstrated clinical efficacy and, moreover, applications for treatment monitoring and control will show a dramatic growth in the next years.

These estimations consistently suggest that liquid biopsy will become an important part of routine clinical laboratory practice, in view of the non-oncological applications of circulating free DNA. Indeed, numerous studies in the last years have explored the diagnostic utility of circulating free DNA in different diseases. Thus, it has shown to be useful in the diagnosis of myocardial infarction, stroke or as a biomarker of organ transplant rejection. It has also been suggested as a prognostic factor in the transplantation of pancreatic islets [9], [10].

Finally, the pre-analytical phase deserves some attention. It is widely known that the reliability of results depends on a careful preanalytical process, and the same occurs with circulating free DNA. In this sense, a multicentre European study investigating the risk of cancer based on DNA concentrations reported large between-centre differences in relation to these concentrations [11]. This finding demonstrates that, as it occurs with other laboratory parameters, preanalytical conditions are critical to obtaining reliable information from liquid biopsy.

In conclusion, the information currently available and future perspectives indicate that the introduction of procedures related to liquid biopsy/circulating free DNA in clinical diagnostics laboratories is a professional challenge that will have to be addressed in the very near future.

## References

[1] Moulierea F, Messaoudia S, Pang D, Dritschilob A, Thierry AR (2014). Multi-marker analysis of circulating cell-free DNA toward personalized medicine for colorectal cancer. Mol Oncol.

[2] Heitzer E, Haque IS, Roberts CES, Speicher MR (2019). Current and future perspectives of liquid biopsies in genomics-driven oncology. Nat Rev Genet.

[3] Lo YM, Corbetta N, Chamberlain PF, Rai V, Sargent IL, Redman CW (1997). Presence of fetal DNA in maternal plasma and serum. Lancet.

[4] Iwarsson E, Jacobsson B, Dagerhamn J, Davidson Bernabé E, Heibert-Arnlind M (2017). Analysis of cell‐free fetal DNA in maternal blood for detection of trisomy 21, 18 and 13 in a general pregnant population and in a high risk population – a systematic review and meta‐analysis. Acta Obstet Gynecol Scand.

[5] Arechederra M, Ávila MA, Berasain C (2020). Liquid biopsy for cancer management. A revolutionary but still limited new tool for precision medicine. Adv Lab Med.

[6] Goodwin S, McPherson JD, McCombie WR (2016). Coming of age: ten years of next-generation sequencing technologies. Nat Rev Genet.

[7] He KY, Ge D, He MM (2017). Big data analytics for genomic medicine. Int J Mol Sci.

[8] Budel S Updated DeciBio NGS report (November 2015).

[9] Snyder TM, Khush KK, Valantine HA, Quake SR (2011). Universal noninvasive detection of solid organ transplant rejection. Proc Natl Acad Sci Unit States Am.

[10] Gala-Lopez BL, Neiman D, Kin T, O’Gorman D, Pepper AR, Malcolm AJ (2018). Beta cell death by cell-free DNA and outcome after clinical islet transplantation. Transplantation.

[11] Gormally E, Hainaut P, Caboux E, Airoldi L, Autrup H, Malaveille C (2004). Amount of DNA in plasma and cancer risk: a prospective study. Int J Cancer.

